# Number of musculoskeletal pain sites leads to increased long-term healthcare contacts and healthcare related costs – a Danish population-based cohort study

**DOI:** 10.1186/s12913-021-06994-0

**Published:** 2021-09-17

**Authors:** S. Mose, P. Kent, A. Smith, J. H. Andersen, D. H. Christiansen

**Affiliations:** 1 Department of Occupational Medicine, University Research Clinic, Danish Ramazzini Centre, Herning, Denmark; 2 VIA University College, School of Physiotherapy, Holstebro, Denmark; 3 Curtin School of Allied Health, Curtin University, Perth, WA Australia; 4 Center for Muscle and Joint Health, University of Southern Denmark, Odense, Denmark

**Keywords:** Number of pain sites, Health anxiety, Healthcare utilization, Musculoskeletal pain, Cohort study

## Abstract

**Background:**

People with musculoskeletal pain seek more healthcare than the general population, however little is known about the long-term effect on healthcare use. The aim of this study was to examine the consequences of number of musculoskeletal pain sites on long-term care-seeking and healthcare-related costs and explore how health anxiety influences this relationship.

**Methods:**

We conducted a Danish population-based longitudinal cohort study of 4883 participants combining self-reported survey data from 2008 with ten-year follow-up data from national health registers. Using a causal inference framework, we examined associations between number of pain sites (range 0–7)/level of health anxiety (high/low level) and face-to-face healthcare contacts/healthcare-related costs. Data were analyzed using negative binomial regression with generalized estimating equations. Regression models were adjusted for sex, age, duration of pain, level of education, comorbidity, personality traits, risk of depression, marital status, physical job exposure, and previous healthcare utilization.

**Results:**

For each additional pain site general healthcare contacts (Incidence Rate Ratio (IRR): 1.04 (95% CI: 1.03–1.05)), healthcare-related costs (IRR: 1.06 (95% CI: 1.03–1.08) and musculoskeletal healthcare contacts (IRR: 1.11 (95% CI:1.09–1.14) increased. Those with high levels of health anxiety at baseline had a slightly higher number of general healthcare contacts (IRR 1.06 (1.01–1.11), independent of number of pain sites. However, level of anxiety did not influence the effect of number of pain sites on any healthcare use or cost outcomes.

**Conclusions:**

We found evidence for a causal association between increasing number of pain sites and greater healthcare use and cost, and high levels of health anxiety did not increase the strength of this association. This suggests that number of pain sites could be a potential target for biopsychosocial interventions in order to reduce the need for future care-seeking.

**Supplementary Information:**

The online version contains supplementary material available at 10.1186/s12913-021-06994-0.

## Background

Musculoskeletal pain is common among adults and one of the most common reasons for care-seeking [1, 2]. People reporting musculoskeletal pain have a higher use of healthcare services than the general population regardless of primary pain site [3]. However, localized pain is relatively rare as musculoskeletal pain often occurs in various body regions simultaneously [4, 5]. This differentiation is important as the functional consequences of pain (sickness absence and disability) increase proportionally with the number of body regions in pain [5–8].

The choice to seek care due to pain has been conceptualized by different theories, with the dominant conceptual framework of health services use being the ‘health behavioral model’ [9]. This model describes care-seeking as a function of individual and contextual predisposing factors, enabling/impeding factors, and perceived need for care [9, 10], driven by an individual’s subjective concerns about health, perception of need and health beliefs rather than an objective, evaluated need [11, 12]. In general, there is high variability in seeking care by different people for the same symptoms, which supports the theory that choice of care-seeking is driven by symptom appraisal [11, 13] and perception of need. Care-seeking could be viewed as a process [12, 14–16] that involves personality, psychological factors and beliefs/knowledge (e.g. fear avoidance, health anxiety, risk perceptions, stress, self-efficacy), comorbidity, type/nature of symptoms/diagnosis (e.g. location, duration, intensity, disability), and social factors [14, 17–20].

Healthcare utilization due to pain and psychological factors, such as health anxiety, has been studied for some years but most research has been undertaken using retrospective or cross-sectional designs of short-term care-seeking from specific healthcare professions or settings (e.g. general practitioner, physiotherapist, emergency department) based on self-reported data, with inherent risks of lag time and recall bias [14, 21]. Therefore, research on care-seeking behavior across professions from a longitudinal long-term perspective was needed. While a number of factors associated with care-seeking for localized pain have been identified in prediction models, few attempts have been made to examine theory-driven causal relationships and underlying determinants of care-seeking behavior for musculoskeletal pain in multiple body sites [14, 17]. Furthermore, knowledge is sparse about the influence of health concerns, such as health anxiety, on the relationship between number of musculoskeletal pain sites and health care utilization.

The aim of this study was to examine the consequences of number of musculoskeletal pain sites on long-term care-seeking and healthcare-related costs and explore whether health anxiety influences this relationship. Our hypotheses were that (i) increasing number of pain sites would result in higher total healthcare utilization and total healthcare-related costs over a subsequent 10-year period, (ii) this relationship would be stronger for people with high levels of health anxiety.

## Methods

### Population

This is a population-based, longitudinal cohort study. In February 2008, web-based and postal questionnaires were sent out to 8517 working age people, registered at the same medical center in the Danish city of Odder staffed by eight General Practitioners (GPs). Overall, 5097 people returned the baseline questionnaire, of whom 4883 were eligible for analysis in the current study. Detailed descriptions of the responders and non-responders have been published elsewhere [19]. In the current study, we excluded patients who were non-identifiable (*n* = 29), died during the 10-year follow-up (*n* = 153) or were living abroad for more than 2 years of the follow-up period (*n* = 32). Participants were between 17 and 65 years of age at baseline. Using validated scales, the questionnaire covered a wide range of information on individual, psychosocial and work-related factors. The following variables were extracted from those baseline questionnaires.

### Exposure (independent variables) – Number of pain sites and Health anxiety

The number of pain sites at baseline was measured using the first part of the pain module of the Standard Evaluation Questionnaire (SEQ) [22]. Participants were asked to state the intensity of pain (if any) on a one (no pain) to seven (worst imaginable pain) point scale in seven different body regions (right/left upper and lower extremity, front and back of thorax and the head) within the last 4 weeks. We classified people as having pain in a body region if they reported pain intensity above 2/7 in that particular region (to exclude trivial pain). This cut-off point is comparable to the optimal cutoff point for a clinical important change on a 0–100 visual analog scale [23, 24]. Number of pain (0–7 sites) sites were analyzed as a continuous variable.

Health anxiety/Illness worry at baseline was measured using the Whiteley-7 Index [25]. Those seven items are included in the Common Mental Disorder Questionnaire (CMDQ). The CMDQ was developed and validated as a case-finding instrument in primary care [26]. This questionnaire contains questions about worries about illness and care-seeking within the last 4 weeks requiring answers using one of five categories from “Not at all” (0) to “Extremely” (4). The 0–28-point score was dichotomized at > 5 (0–5 = low risk, 6–28 = high risk) as the threshold of scores above five is recommended by The Danish College of General Practitioners as an indication of clinically relevant risk of health anxiety [27]. The Whiteley-7 Index has shown acceptable psychometric properties in primary care settings [25].

### Co-variables derived from baseline questionnaire

To capture the five basic dimensions of personality (Extraversion, Agreeableness, Conscientiousness, Neuroticism and Openness) we used the five-factor model of personality traits, derived from 20 items in the Mini International Personality Item Pool (IPIP) which is a validated tool for measuring personality traits [28, 29]. In the mini-IPIP, each personality trait is measured with four items. This questionnaire asks participants to state their level of agreement with statements like “I am the life of the party”, “I have frequent mood swings”, “I sympathize with others’ feelings” in five categories from “Very Inaccurate” to “Very Accurate”. Each personality trait scale was summed to a 0–16 score and analyzed as a continuous variable.

Risk of depression at baseline was measured using six items (SCL_DEP6) from the Symptom Checklist 90-items. These items are included in the Common Mental Disorder Questionnaire (CMDQ). The CMDQ was developed and validated as a case-finding instrument in primary care [26]. This questionnaire contains six questions about influence of negative mood and feelings within the last 4 weeks and requires answers using one of five categories from “Not at all” to “Extremely”. We dichotomized the 0–24-point score at five (no risk of depression/risk of depression) based on recommendation from The Danish College of General Practitioners as scores above five indicate enlarged risk of depression [27].

Duration of pain at baseline was measured with a single question item from part 4D of SEQ [22]. Participants were asked to nominate the duration of their pain using one of the following four categories: “Less than a month”, “One to three months”, “Four to twelve months” and “More than a year”. For the purpose of this study, duration of pain at baseline was dichotomized into ‘no chronic pain’ for participants reporting pain for less than 3 months and ‘chronic pain’ for participants reporting pain for more than 3 months. As this question was asked in a way where people without pain were left with no obvious choice, we decided to code missing on this item as ‘no chronic pain’ for those who reported no pain in any body region. Furthermore, we included participants who reported no pain in any body region within the last 4 weeks into the ‘no chronic pain’ group.

### Variables derived from National registers

Using the civil registration number uniquely assigned to each resident of Denmark, baseline data was linked to Danish health and social registers to extract data for the subsequent 10-year period (2008–2017) [30, 31]. Number of healthcare contacts and related costs were based on information from the National Health Insurance Service Register [32], the National Patient Register [33, 34], the Register of Medicinal Product Statistics [35], the Rehabilitation According to “The Danish Act of Health §140” register and Diagnosed Related Grouped National Patient Register Data. Information about education level, death and migration was obtained from the Population Education Register and Population Statistics Registers.

The National Health Insurance Service Register (NHSR) was established in 1990 and contains information about all fully or partially public funded primary healthcare services. The NHSR contains information about the healthcare provider, public expenditure for each contact and the type of service provided based on the week reimbursement was claimed for [32, 36]. The diagnosis or reason for the consultation is not recorded in the NHSR. A minor proportion of primary care physiotherapy and chiropractor consultations are fully self-funded, and therefore not recorded in the NHSR. This proportion of physiotherapy and chiropractor healthcare has been estimated to about 15% by the Danish Physiotherapist Association.

The National Patient Register (NPR) is the central register for recording activity in the Danish secondary healthcare system. The NPR contains information on hospital admissions since 1978 and all outpatient hospital contacts since 1994. Information in the NPS includes the date and time for hospital arrival and departure, type of contact, diagnosis, treatment and tests. Registration in the NPS is based on the Healthcare Classification System [37] and diagnostic criteria are the International Statistical Classification of Diseases and Related Health Problems 10th Revision (ICD-10) diagnostic codes. In general, data from the NPS are considered valid, but the positive predictive values of diagnostic codes can vary for different diseases and types of treatment [34]. In this study, information from the NPR was used to identify number of contacts and reason for contact.

The Diagnoses Related Group (DRG) Grouped National Patient Register covers data about costs related to in- and out-patient contacts in the NPR. DRG-grouped NPR data itemize payment instances and rates in the Danish healthcare system. Estimated grouped rates for each hospital service are based on the average costs for all hospitals in Denmark and are used for government payments to Danish hospitals. The payment rates for DRG-grouped NPR items are re-evaluated each year.

The Rehabilitation According to “The Danish Act of Health §140” register (Rehab Register) was established in 2007. According to Danish law, after hospital admission or outpatient encounters, patients can be referred to publicly funded physiotherapy and occupational therapy rehabilitation if medically indicated. The Rehab Register contains information about contact dates and number of contacts during this type of rehabilitation.

Data on sex and age was obtained from the Danish Civil Registration System [30, 31, 38]. Age was analyzed in six ordinal groups (17–20, 21–30, 31–40, 41–50, 51–60 and 61–65 years of age).

Highest achieved level of education at each follow-up year was obtained from The Danish Education Register [39]. Classification was based on ‘The International Standard Classification of Education’ [40] and categorized into three ordinal groups: 1) primary and lower secondary education or equivalent, 2) upper secondary education or skilled worker/short cycle tertiary education or equivalent, and 3) Bachelor/Master/Doctorial or equivalent. Level of education was analyzed longitudinally, hence, participants were able to change education group during follow-up.

Comorbidity was obtained by applying an updated version of Charlson comorbidity index to ICD10 diagnostic codes in NPR [41]. The Charlson comorbidity index is a valid prognostic indicator for mortality in various disease subgroups and the index has been widely used as an indicator for comorbidity in epidemiology and clinical research, including research on pain and pain-related outcomes [41]. Using ICD-10 diagnostic codes to ascertain the Charlson comorbidity conditions in NPR data has shown high accuracy [42]. A higher Charlson comorbidity score indicates an increased amounts of comorbidity [43]. As data on comorbidity was zero inflated, we categorized the comorbidity index into three ordinal groups (0 - no comorbidity, 1 - low level of comorbidity and > =2 - high level of comorbidity). Comorbidity was analyzed longitudinally. Baseline index was calculated using the previous 2 years of NPS data and thereafter the index was updated each follow-up year; hence the comorbidity index across the whole study period was based on 12 years of NPS data.

Physical lower body occupational job exposure information was obtained by linking the Danish version of the International Standard Classification of Occupations (D-ISCO 88) job titles from the Register-Based Labour Force Statistics [44, 45] to the Lower Body Job Exposure Matrix (JEM) [46]. The lower body JEM estimates lower body occupational exposure based on five experts’ ratings from 121 occupational groups including 689 occupational titles with similar exposure. For this study we used the variable ‘total kilograms (kg) lifted per day’ dichotomized into < 1000 kg/day (low) and ≥ 1000 kg/day (high) [47]. Similarly, we linked D-ISCO 88 job titles to the shoulder JEM [48, 49]. To estimate physical occupational shoulder exposure we used the total shoulder score (range 0–10), which is a combined measure of seven different shoulder exposures (upper arm elevation > 90°, repetitive shoulder movements, forceful shoulder exertions, lifting/carrying, and pushing/pulling, use of handheld vibrating tools and computer work) [49], and dichotomized that score into high and low scores at the 75th percentile. Most participants had follow-up years with missing or incomplete D-ISCO 88 codes, meaning that physical job exposure could not be calculated for all follow-up years. If possible, we carried forward previous years D-ISCO 88 code if information from “The Danish Register-based Evaluation of Marginalized Individuals” (DREAM-register) [50] indicated that the participants had been working more than 26 weeks that year.

Marital status and number of resident children under the age of eighteen was obtained from the Danish Civil Registration System [30]. We combined data on marital status and number of resident children under the age of eighteen into the following four categories: 1) Cohabitant with resident child/children, 2) Cohabitant without resident children, 3) Single with resident child/children, and 4) Single without resident children. Marital status was analyzed longitudinally, hence, participants were able to change status during follow-up.

Use of healthcare the last 2 years before baseline was derived by applying the procedure and algorithm for the dependent variable (Table 1 and Appendix A (see Additional file 1)) on 2006 and 2007 data from NPS, NHSR and the Rehab-register. Musculoskeletal-related healthcare contacts and all healthcare contacts for these years were summed separately and analyzed as a continuous variable.

**Table 1:** Outcome (dependent variables) - healthcare contacts and healthcare-related costs

	Register	Method
Number of **musculoskeletal healthcare contacts (MSK-contacts)**:	NPR	1) Counts of in- and out-patient hospital contacts and emergency department contacts registered with a primary or secondary musculoskeletal or pain-related ICD-10 diagnostic code. Every inpatient admission day counted as one contact. See Appendix A (Additional file 1). for more detail.
	NHSR	2) Counts of face-to-face primary care consultations with physiotherapists, chiropractors and musculoskeletal medical specialists. Excluded in this category was fully publicly reimbursed encounters with physiotherapists for non-musculoskeletal diagnoses.
	NHSR	3) Counts of face-to-face GP contact where the clinical tests, examination, coding and subsequent healthcare initiatives indicated a musculoskeletal reason for that consultation. For this purpose, a simple algorithm was developed. The algorithm evaluated each face-to-face GP contact in two steps and built on available information from all health registers. For a more detailed description, see Appendix B (Additional file 2) for more detail. Validation of this algorithm is pending.
	Rehab-register	4) Counts of face-to-face municipality musculoskeletal rehabilitation visits indicated by a prior musculoskeletal hospital in- or out-patient contact.
N**umber of healthcare contacts for any reason (All-contacts)**:	NPR	1) Counts of all in- and out-patient hospital contacts and emergency department contacts without regard for ICD-10 diagnostic codes. Each inpatient admission day counted as one contact. Derived from the NPR.
	NHSR	2) Counts of all face-to-face primary care physiotherapy, chiropractic, podiatrist/chiropodist, psychologist, dentist and medical specialist consultations.
	NHSR	3) Counts of all face-to-face GP consultations.
	Rehab-register	4) Counts of all municipality rehabilitation consultations.
**Costs related to all healthcare contacts (All-costs)**:	DRG/NPR	1) DRG-cost from all in- and out-patient hospital contacts and emergency department contacts.
	NHSR	2) Public expenditure for all primary care physiotherapy, chiropractic, podiatrists/chiropodist, psychologist, dentist, GP and medical specialist consultations.
	Rehab-register	3) Calculated expense based on salary and other operating costs for all municipality rehabilitation settings.

### Outcome (dependent variables) - healthcare contacts and healthcare-related costs

For the purpose of this study, two categories of outcomes were derived from these healthcare registers: the number of face-to-face contacts, and healthcare costs. Number of face-to-face contacts were derived separately for musculoskeletal healthcare (MSK-contacts) and for healthcare contacts for any reason (All-contacts). These two outcomes were derived by counting healthcare contacts per participant for each follow-up year (2008 to 2017) in the HSR, NPR and Rehab registers. Details for this procedure are displayed in Table 1 and Appendix A (see Additional file 1). Costs related to healthcare contacts (All-costs) per participant for each follow-up year was derived by summarizing DRG-costs, public expenditure for all primary care contacts and estimated expenditure for rehabilitation contacts. Details are presented in Table 1 and Appendix A (see Additional file 1). Due to the data structure in the DRG and the LPR it was not possible to match secondary healthcare cost and MSK-contacts with satisfactory precision; hence MSK related costs were not be derived. All-costs are presented in Euro (€).

### Statistical analysis

This study is based on a causal inference framework [51–53]. Each research question had its own theoretical framework which guided analysis and hypothesis testing. This framework was based on our interpretation of previous literature in this field and has been visualized using Directed Acyclic Graphs (DAGs) (www.dagitty.net) [54] (Appendix C) (see Additional file 3). The choice of co-variables for statistical adjustment for each research question has been guided by the principle of minimal sufficient adjustment sets of co-variables for estimating the total effect [54, 55].

To describe the sample, we used frequencies and percentages for categorical and dichotomous variables and means/medians with standard deviations or 25th /75th percentiles for continuous variables. To understand the relationship between key variables, the correlation between number of pain sites and health anxiety, as well as between the outcomes of costs and counts (MSK-contacts, All-contacts and All-costs), were tested with Spearman’s correlation coefficients with 95% CI estimated using bootstrapping methods with 100 repetitions. Each hypothesis was tested via a negative binomial distribution regression model using Generalized Estimating Equations (GEE) to account for multiple observations on the same person over the study time periods and right skewed zero inflated count data (proportional differences in means accounting for zero-inflated data). We calculated adjusted incidence rate ratios (IRR) for the total effect between the independent variable (either pain areas or health anxiety) and healthcare visits or related costs with 95% CI. We tested for an interaction between number of pain sites and health anxiety in each outcome model. The choice of variable, and hence adjustment for each hypothesis test, was informed by theoretical considerations and DAGs (Appendix C (see Additional file 3)). Regression models were adjusted for sex, age, duration of pain, level of education, comorbidity, personality traits (extraversion, agreeableness, conscientiousness, neuroticism and openness), risk of depression, marital status, physical job exposure and previous healthcare utilization. Decisions about the correlation structure for GEE were informed by visual inspection and by Quasi-likelihood under Independence model Criterion test. Based on this evaluation, an unstructured correlation structure was chosen. To optimize the adjustment, we allowed for an interaction between sex and age within the adjustment of all three models.

All variables based on baseline questionnaire data contained some missingness (usually 3–5%) and some D-ISCO 88 codes were missing for one or more follow-up years. This was managed with chained multiple imputation techniques imputing ten datasets to account for the uncertainty in the imputation. As a sensitivity analysis, estimates from the primary regression analysis on multiple imputation data were compared with estimates from a full case analysis on non-imputed data. Only results based on multiple imputation data will be presented. Chained multiple imputation was applied on 1187 cases missing baseline variables or physical job exposure. Twenty-seven percent of these cases had missing values on one variable, 49% on two variables and 24% on three or more variables. Most missing values were on physical job exposure (54%).

All statistics were performed using STATA (College Station, Tx, USA). As advised by Bland and Altman [56], two-sided statistical tests were used despite the presence of directional hypotheses.

## Results

### Baseline characteristics

The characteristics of included participants are presented in Table 2. Twenty-nine percent of the participants reported no pain at any site at baseline, whereas participants reporting pain in five or more body sites were relatively rare (12% in total). Low level of health anxiety at baseline was reported by 80% of the participants. Fifty-six percent of the population were women and the mean age at baseline was 45 years (SD 12.8) with the largest age group being 50–60-year old’s (28%). Most participants with pain reported chronic pain. Approximately one third of the study population reported pain for less than 3 months (36%, *n* = 1669). The vast majority of participants (95%) had no comorbidity at baseline. At baseline, 46% of the participants were living with a partner and had children residing with them. Most of the participants had low level of physical job exposure at baseline (Lower body: 87%. Upper body: 72%).

**Table 2:** Baseline characteristics by total number of healthcare contacts and total health care costs (2008 to 2017)

	Total, n (%)	Total number of All-contacts(a)	Total All-cost(a)	Total number of MSK-contacts(a)
		Median (25;75 percentile)	Median (25;75 percentile)	Median (25;75 percentile)
**Total, median (25;75 percentile)**	4883 (100)	74 (42;124)	€197 (84;611)	11 (2;33)
**Number of sites with pain intensity > 2/7 (b)**				
**0, n (%)**	1365 (29)	55 (33;92)	€144 (67; 387)	6 (1;17)
**1, n (%)**	914 (19)	67 (38;109)	€160 (71;472)	9 (1;27
**2, n (%)**	810 (17)	77 (44;119)	€220 (101;684)	11 (3;34)
**3, n (%)**	642 (14)	87 (52;143)	€239 (105;636)	18 (5;43)
**4, n (%)**	464 (10)	105 (59;167)	€276 (118;910)	21 (7;54)
**5, n (%)**	265 (6)	105 (69;163)	€361 (132;991)	25 (10;53)
**6, n (%)**	175 (4)	125 (62;205)	€447 (143;1330)	31 (10;65)
**7, n (%)**	75 (2)	150 (87;209)	€331 (181;1581)	28 (15;67)
**Health anxiety(c)**				
**Low, n (%)**	3811 (80)	68 (38;112)	€171 (78;487)	10 (2;28)
**High, n (%)**	933 (20)	104 (62;177)	€365 (141;1068)	20 (6;50)
**Covariates**				
**Sex**				
**Female, n (%)**	2735 (56)	87 (54;140)	€253 (106;752)	15 (4;38)
**Male, n (%)**	2148 (44)	57 (31;101)	€150 (64;409)	8 (1;25)
**groups, baseline, mean (sd)**	45 (12.8)			
**17–20, n (%)**	301 (6)	53 (29;93)	€100 (46;246)	4 (1;14)
**21–30, n (%)**	399 (8)	68 (40;112)	€201 (71;827)	8 (1;21)
**31–40, n (%)**	918 (19)	59 (34;101)	€162 (67;527)	11 (2;32)
**41–50, n (%)**	1309 (27)	68 (39;111)	€180 (77;486)	13 (3;37)
**51–60, n (%)**	1389 (28)	86 (49;138)	€231 (108;705)	14 (3;36)
**61–65, n (%)**	567 (12)	105 (69;163)	€312 (121;889)	14 (4;37)
**Duration of pain(d)**				
**No chronic pain, n (%)**	1669 (36)	62 (36;103)	€165 (78; 512)	7 (1;22)
**Chronic pain (> 3 month), n (%)**	2941 (64)	82 (46;137)	€219 (91;656)	15 (4;39)
**Educational level(e)**				
**Primary and lower secondary education, n (%)**	992 (20)	75 (42;130)	€188 (78;535)	9 (2;28)
**Upper secondary education or Skilled worker, n (%)**	2570 (53)	76 (43;127)	€203 (86;628)	13 (3;36)
**Bachelor/Master/Doctorial, n (%)**	1301 (27)	70 (40;114)	€190 (87;620)	10 (2;28)
**Comobidity**				
**No cormorbidity, n (%)**	4638 (95)	71 (41;119)	€181 (81;508)	11 (2;31)
**Low level of comorbidity, n (%)**	137 (3)	177 (102;274)	€1167 (451;2966)	32 (10;75)
**High level of comorbidity, n (%)**	108 (2)	149 (80;256)	€1318 (494;3080)	15 (5;34)
**Big five personality traits(f) (range 0–16)**				
**Neuroticism, mean (sd)**	6.5 (3.0)			
**Extraversion, mean (sd)**	8.6 (3.1)			
**Agreeableness, mean (sd)**	11.6 (2.2)			
**Conscientiousness, mean (sd)**	10.7 (3.0)			
**Openness, mean (sd)**	9.1 (3.1)			
**Risk of depression (g)**				
**Low, n (%)**	3982 (84)	69 (40;116)	€181 (81;533)	10 (2;30)
**High, n (%)**	772 (16)	100 (56;174)	€315 (116;873)	17 (5;45)
**Marital Status**				
**Cohabitant with resident children, n (%)**	2267 (46)	61 (36;105)	€158 (73;469)	10 (2;29)
**Cohabitant without resident children, n (%)**	1726 (35)	88 (51;149)	€257 (108;791)	14 (3;36)
**Single with resident children, n (%)**	272 (6)	80 (46;132)	€251 (96;658)	12 (3;40)
**Single without resident children, n (%)**	618 (13)	82 (42;135)	€196 (78;602)	11 (2;30)
**Physical job exposure**				
**Total kilograms lifted (lower body) (h)**				
**< 1000 kg, n (%)**	3807 (87)	71 (41;119)	€186 (82;576)	11 (2;32)
**> 1000 kg, n (%)**	551 (13)	68 (36;113)	€164 (73;444)	11 (2;32)
**Total shoulder score (upper body) (i)**				
**Low, n (%)**	3142 (72)	74 (42;125)	€197 (86;626)	11 (2;33)
**High, n (%)**	1220 (28)	63 (36;105)	€161 (71;431)	10 (2;29)

a: 2008–2017. b: Missing: *n* = 173. c: Missing: *n* = 139. d: Missing: *n* = 273. e: Missing: *n* = 20. f: Missing: *n* = 198–240. g: Missing: *n* = 129. h: Missing: *n* = 525. i: Missing = 521

### Number of healthcare contacts and costs

Women and participants above 50 years of age at baseline had higher median number, total All-contacts, total ALL-costs and total MSK-contacts compared to men and participants < 50 years of age at baseline. The median number of total ALL-contacts, total ALL-costs and total MSK-contacts increased with increasing number pain sites, high level of health anxiety, duration of pain and high risk of depression. For level of co-morbidity, educational level, marital status and physical job exposure this pattern was inconsistent (see Table 2).

The correlation between annual number of MSK-contacts and All-contacts was 0.60 (95% CI 0.59–0.61), between MSK-contacts and All-costs was 0.44 (95% CI 0.43–0.45), and between All-contacts and All-costs was 0.85 (95% CI 0.84–0.85). Correlation between the number of pain sites and health anxiety was 0.39 (95% CI 0.38–0.40).

Based on the theoretical model displayed in Appendix C (see Additional file 3), is was estimated that the consequence of each additional pain site was an increase in long term healthcare-seeking for All-contacts (IRR: 1.04 (95% CI: 1.03–1.05)), All-costs (IRR: 1.06 (95% CI: 1.03–1.08)) and MSK-contacts (IRR: 1.11 (95% CI:1.09–1.14)). Testing the same hypotheses with number of pain sites as an ordinal variable showed that this incremental increases in healthcare utilization were the same for each additional pain site (data not shown). This means that a person reporting no pain at baseline has an adjusted mean number of 8 (95% CI: 7.7–8.2) All-contacts, All-costs of € 895 (95% CI: 844–948) and 1.7 (95% CI: 1.6–1.8) MSK-contacts per follow up year, whereas the corresponding values for a person reporting pain in seven body sites are: 10.3 (95% CI: 9.7–11) All- contacts, € 1324 (95% CI: 1178–1489) All-costs and 3.6 (95% CI: 3.2–4) per follow up year.

Independent of number of pain sites, high level of health anxiety at baseline resulted in a slight increase in All-contacts (IRR:1.06 (95% CI: 1.01–1.11)) and All-costs (IRR: 1.09 (95% CI: 0.99–1.20)) (Table 3). This increase was only statistically significant for All-contacts. However, there was no evidence that low or high health anxiety influenced the effect of number of pain sites on any healthcare utilization outcomes (data not shown).

**Table 3:** Associations between number of pain sites/health anxiety and number of healthcare contacts for any reason (All-contacts), healthcare-related costs (All-costs) and number of musculoskeletal healthcare (MSK-contacts)

***N*** = 4883	All health care seeking		Musculoskeletal health care seeking
Variable	Number of contacts(All-contacts) Adjusted IRR (95% CI)	Costs (All-costs) Adjusted IRR (95% CI)	Number of contacts (MSK-contacts) Adjusted IRR (95% CI)
Number of pain sites at baseline with pain intensity> 2/7 (0–7) (a)	1.04 (1.03–1.05)	1.06 (1.03–1.08)	1.11 (1.09–1.14)
Health anxiety (a)			
- Low score - High score	Ref- 1.06 (1.01–1.11)	Ref- 1.09 (0.99–1.20)	Ref- 1.02 (0.92–1.12)

Detailed description and footnotes

a: Adjusted for: Sex, age, duration of pain, level of education, comorbidity, personality traits (extraversion, agreeableness, conscientiousness, neuroticism and openness), risk of depression, marital status, physical job exposure and previous healthcare utilization

**Fig. 1:**
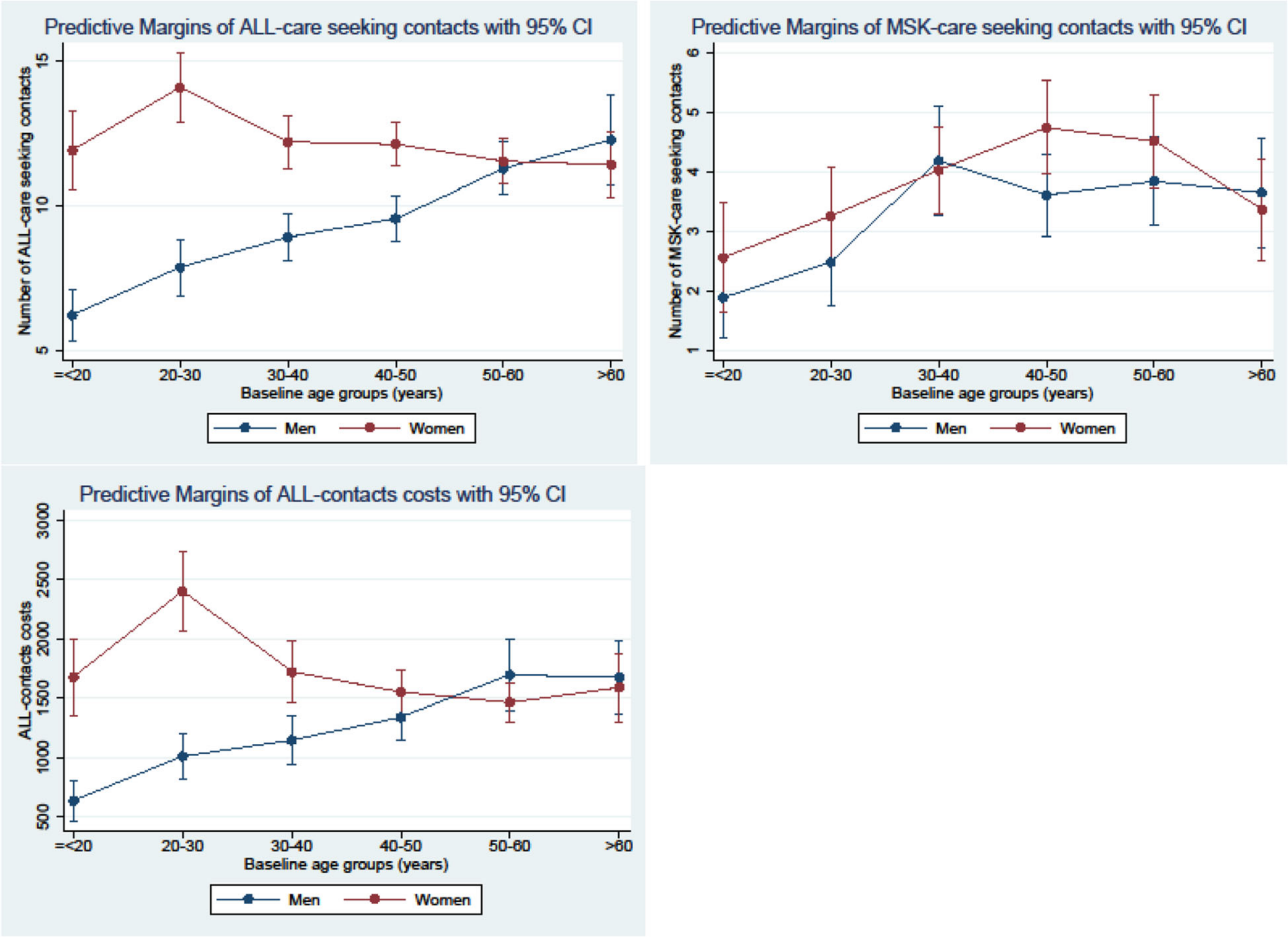
Predictive margins of All-care-seeking, MSK-care-seeking and All-contacts costs for baseline age-groups for men and women

Allowing for different effect of sex and age in the adjustment model revealed that females below the age of 50 had more All-contacts and higher All-costs than age-matched males. This difference was not evident for MSK-contacts (Fig. 1). Estimates from analysis on multiple imputation data and non-imputation data were similar (only results from analysis on multiple imputed data are shown).

## Discussion

### Main results

Based on a causal inference framework, this population-based cohort study found that for every additional pain site a participant reported at baseline, over the subsequent 10 years their healthcare-contacts for any reason, healthcare-related costs and musculoskeletal-related healthcare contacts increased. Non-overlapping confidence intervals for these estimates show that this increase was highest for musculoskeletal-related contacts (11%). Independently of number of pain sites, adults with high level of health anxiety at baseline had more healthcare-contact and higher healthcare-related costs compared to people with low level health anxiety, but this association was weak. Additionally, we found no evidence that level of health anxiety (high vs low) influenced the effect of number of pain sites on healthcare utilization. However, we did find that females below the age of 50 had more healthcare-contacts for any reason and higher healthcare-related costs than age-matched males.

### Strengths and limitations

This study has several strengths. The prospective, population-based cohort design ensured that information about exposures and co-variables were collected prior to, and independently, of the outcome, which prevents differential misclassification. By linking national health registers and administrative outcomes data to self-reported baseline survey data via personal identification numbers, we insured person-level complete data linkage of healthcare contacts and costs in both the primary and the secondary healthcare sector over a ten-year follow-up period. The validity and completeness of Danish national healthcare register data for number of contacts is considered high [32, 34, 57].

However, some limitations should be noted. As the NHSR database does not contain information on diagnostic coding, MSK-contacts in primary health were based on information about professional groups e.g. physiotherapists, chiropractors and orthopedic surgeons, where their scope of practice indicates that any consultations likely relate to musculoskeletal complaints. For GP contacts, we developed an algorithm to identify MSK-specific contacts (Appendix B (Additional file 2)). This approach may have led to some misclassification but similar approaches have been applied and validated using NHSR data for linking patients and general practices with promising results [58]. Based on self-reported data, about 78% of Danish adults consult their GP each year [59]. Approximately 14% of these consultations are related to musculoskeletal disorders [60, 61]. Our algorithm estimated 18% (95% CI 18–19%) of face-to-face GP consultations to be MSK-related, which is slightly higher than those previous studies but still credible. Another limitation with the NHSR database is that approximately 15% of chiropractors and physiotherapist consultations in primary care are payed either fully out-of-pocket by patients or insurance without any public reimbursement and hence not reported to the NHRS [62]. These consultations could therefore not be accounted for in our investigation. Furthermore, all secondary MSK healthcare contacts were based on ICD-10 diagnostic codes in the NPS database and the positive predictive values of diagnostic coding may vary. Nonetheless, the validity and completeness of the NPR database are considered to be relatively high [34]. The recoding of the baseline question about duration of pain may have led to some misclassification but sensitivity analysis indicates that this approach had no influence on the main results.

Exposure variables were only measured at baseline. However, previous research has shown that pain sites and prevalence are relatively stable phenomena [63]. Kamaleri et al. found that number of pain sites change relatively little over a 14-year follow-up period [64] and Paananen et al. found that 75% of boys and 88% of girls with widespread pain at age 16 subsequently reported a similar pain pattern at age 18 [65]. In both studies, only a few percent of participants with baseline pain reported no pain at follow-up. Likewise, health related concerns, such as health anxiety, are also considered a persistent trait despite reassurance [66, 67]. However, data quality would have been increased had we had repeated measures over time of pain sites.

We dichotomized the Whiteley-7 Index, while others have analyzed this scale as a categorical or continuous variable. The choice to dichotomize was guided by recommendations for clinical use [25, 27] and therefore the findings may be more relevant to clinical practice. Similarly, risk of depression and physical work exposure were dichotomized based on recommendations and former use of these scales, and comorbidity index were generated based on secondary health care data only, introducing a risk of residual confounding. Lastly, only 4883 (57%) of eligible participants responded to the baseline questionnaire, and while a description of non-participants has been published elsewhere [19], additional data on non-participants were not available. While such modest participation rates are not uncommon in large population studies, we cannot rule out the potential for some unquantified selection bias. However, simulation studies have shown that modest participation rates do not necessarily affect estimated associations between variables [68].

### Results in light of theory and research literature

Only about one third of people with musculoskeletal pain seek care because of their pain [13] and the decision to seek care is influenced by a range of factors [9, 14, 21]. We chose to build our causal model with a primary focus on number of pain sites and explored the influence of health anxiety. Both factors have been identified as prognostic factors for care-seeking but, to our knowledge, no previous study has tested causal hypotheses about the consequences of these factors on healthcare-seeking and related costs.

Co-occurrence of musculoskeletal pain in different body regions are common [5, 7] and people reporting musculoskeletal pain also report comorbidities, and other symptoms than pain, more frequently than people without pain [6, 69, 70]. This suggests that musculoskeletal pain may be an indicator of poor general health and hence increased general healthcare utilization. Still, our findings show that pain in more body sites leads to a higher increase in long term musculoskeletal healthcare-seeking than general healthcare-seeking suggesting that general healthcare-seeking is different and potentially has different drivers. Relatively few in this sample had any comorbid diagnosis (95% of the sample have no comorbidity at baseline). Comorbidity was measured with Charlson comorbidity index on NPS data. Even though such approach is considered valid [42] it is likely an underestimation of comorbidity compared to self-reported comorbidity as we applied the Charlson comorbidity index algorithm on NPS data searching for only hospital verified ICD-10 diagnostic codes given in a period of no more than 2 years.

A priori we had anticipated that contacts and costs might show different results, as some contacts have significantly higher costs (e.g. in-patient hospital contacts or surgery), but number of pain sites show quite similar associations between All-contacts and All-costs. This is understandable given the high correlation (0.85) between these outcomes and indicate that general healthcare utilization is similarly estimated by either method.

Previous studies have found that health reassurance-seeking is prevalent among individuals with high levels of health anxiety and they tend to make stronger requests to healthcare professionals for expensive diagnostic tests and unnecessary treatments [71]. We found no interaction between number of pain sites and health anxiety on any of the outcomes, and the correlation between health anxiety and number of pain sites in this study was low which indicates that health anxiety and number of pain sites appear to act independently.

In designing this study, we took the position that increasing number of pain sites leads to more healthcare-seeking and costs and that this mechanism works through pathways of factors, such as catastrophizing, fear avoidance beliefs and health-related quality of life. This position aligns with behavioral models, e.g. the ‘fear avoidance model’ [72] or ‘the common sense model’ [73]. Adjustment in this study was based on ‘minimum set of confounders’ to estimate the total effect of each exposure on each outcome. Our variable selection and adjustment were informed by literature, theory and discussions between the authors and our hypothetical causal models were illustrated in directed acyclic graphs (Appendix C (see Additional file 3)). This approach was guided by the recommendations from 47 journal editors for control of confounding and reporting of results in causal inference studies [52].

### Care-seeking and healthcare costs

In most Western countries, care-seeking has gained increasing attention as the prevalence of pain and healthcare-related costs has increased during the past two decades [74, 75]. One possibility is that the increase in care-seeking is the result of healthcare overuse (defined as “the provision of medical services that are more likely to cause harm than good” [74]). Examples of overuse are unnecessary tests with the detection of unimportant findings or redefining boundaries for disease that result in more healthcare treatment with little or no net benefit [75].

The purpose of this study was not to analyse if increased healthcare use with increased pain sites is helpful, or a result of healthcare overuse or if it is guideline-adherent and evidence based. However, such topics are important for future projects. Instead these results provide insight into the relationship between number of musculoskeletal pain sites, health anxiety and healthcare utilization and highlights factors that may contribute to non-guideline adherent clinical pathways.

## Conclusion

Our findings show that increasing number of pain sites is associated with higher number of general healthcare contacts, higher healthcare-related costs and higher number of musculoskeletal healthcare contacts over a subsequent ten-year period. We found a weak association between health anxiety and higher number of general healthcare contacts and no evidence that level of health anxiety influences the effect of number of pain sites on healthcare utilization outcomes. In this context, our results add knowledge about drivers of care-seeking and may assist healthcare professionals in formulating patient communication and clinical decision-making in order to optimize healthcare utilization. This study is also a step towards better understanding of a population of patients that might not benefit from current clinical pathways and the organization of healthcare systems in most Western countries. The comorbid nature of pain characterized by multiple pain sites calls for comprehensive collaboration across disciplines which can be a challenge within the silo-organization of most healthcare systems and healthcare sectors. In order better embrace this population of patients in the healthcare system and avoid healthcare overuse, we need more knowledge about the healthcare pain management trajectories across sectors and disciplines. Such knowledge could potentially highlight management gaps or specific patient groups in high risk of non-guideline-adherent clinical pathways. Another important knowledge gap for future research projects of health care service use is the perspective of the health care user. Such knowledge could also guide healthcare providers in their communication with people with multi-site pain in order optimize patient-centered healthcare pain management.

## Supplementary Information

**Additional file 1.**

**Additional file 2.**

**Additional file 3.**

## Data Availability

Data from Danish National Registers are available from the Danish National Health and Medicines Authority for researchers who meet the criteria for getting access to micro data. According to Danish regulations, researchers who are interested can only apply for access through an affiliation to a Danish authorized research environment and apply for data access directly at Statistics Denmark (https://www.dst.dk/en/TilSalg/Forskningsservice) and The Danish Data Protection Agency (https://www.datatilsynet.dk/english). Interested researchers may contact the corresponding author of this article for further guidance on this procedure.

## Abbreviations

GP: General Practitioners
NHSR: The National Health Insurance Service Register
NPR: The National Patient Register
DRG: The Diagnoses Related Group Grouped National Patient Register
Rehab Register: The Rehabilitation According to “The Danish Act of Health §140” register
CMDQ: International Classification of Diseases and Related Health Problems version 10 - ICD10. Common Mental Disorder Questionnaire
SEQ: the Standard Evaluation Questionnaire
IPIP: The Mini International Personality Item Pool
D-ISCO 88: International Standard Classification of Occupations
JEM: Lower Body Job Exposure Matrix
DREAM-register: Danish Register-based Evaluation of Marginalized Individuals
MSK-contacts: Musculoskeletal healthcare contacts
All-contacts: Healthcare contacts for any reason
Cost: Costs related to all healthcare contacts
DAGs: Directed Acyclic Graphs
GEE: Generalized Estimating Equations
IRR: Incidence rate ratios
