# Supplementary material for: Number of musculoskeletal pain sites leads to increased long-term healthcare contacts and healthcare related costs – a Danish population-based cohort study
Source: BMC Health Serv Res. 2021 Sep 17;21:980. doi: 10.1186/s12913-021-06994-0 (PMC8447684; doi:10.1186/s12913-021-06994-0)
Supplement: Supplementary file 1 — Additional file 1. [file 12913_2021_6994_MOESM1_ESM.docx]

**Appendix A: Data management plan for deriving healthcare contacts and healthcare-related costs from The National Patient Register (NPR), The National Health Insurance Service Register (HISR), The Rehabilitation According to “The Danish Act of Health §140” register (Rehab-register) and The Diagnoses Related Group (DRG) Grouped National Patient Register.**

| **DATAMANAGEMENT - Health registers** | | | |
| --- | --- | --- | --- |
| **Registre** | **Definitions** | **Annual number of contacts (2006 – 2017) for each participant based on counts of:** | **Annual health care costs (2006 – 2017) for each participant based on:** |
| **The National Patient Register (NPR)** | Contacts will be defined as a registered date with a health care encounter (out-patient visits (e.g. test, surgery, treatment), inpatient or emergency department visits). Each course may have several contacts but only one contact each day will be considered (e.g. examination, imaging and surgery on the same day, will be considered as one contact). Inpatient encounters will be considered as one contact per day. | **Primary (A) and secondary (B) diagnosis.**   **1. Number of All face-to-face contacts:** All contacts registered with any ICD10 code  **2.** **Number of Musculoskeletal face-to-face contacts**:  M (Chapter XIII - Diseases of the musculoskeletal system and connective tissue ) - All codes.  G (Chapter VI Diseases of the nervous system) - Following codes: G43 (migraine), G44 (headache), G546+547 (phantom pain), G500A+501 (facial pain), G55 + G56 + G57 (nerve compression from discus/stenosis or in UE/LE)    R (Chapter XVIII - Symptoms, signs and abnormal clinical and laboratory findings, not elsewhere classified) - Following codes: R52 (nonspecific pain syndrome), R51 (Headache)  S (Chapter XIX - Injury, poisoning and certain other consequences of external causes) - Following codes:  S12+13+16 (neck), S22+23 (Thorax), S32+33 (Low back/pelvis), S42+43+46 (Shoulder/Upper arm), S52+53+56(elbow/lower arm), S62+63+66(Hand) S72+73+76(Hip/thigh), S82+83+86(Knee/crus), S92+93+96(Ankle/foot), T02+03+06(Multiple)  K (Chapter XI - Diseases of the digestive system) - Following code: K076A (mandibular joint pain) | No cost data in NPR. |
| **The National Health Insurance Service Register (HISR)** | Contacts will be defined as a health-related or medical service face-to-face encounter. E-mail and telephone contacts will not be considered. NISR service codes chosen to represent face-to-face health care contacts are based on agreements between The Danish health Authority and relevant professional organizations (see [www.okportalen.dk](http://www.okportalen.dk)). | **1. Number of all primary health care face-to-face contacts:**  Anesthesiology (Spec. 01), Diagnostic radiology (Spec 03 + 05), Dermatology (Spec 04), Rheumatology (Spec 06), Gynecologist (Spec 07), Internal medicine (Spec 08), Surgery (Spec 09), Neuro-medicine (Spec 18), Ophthalmologist (Spec 19) Orthopedic surgery (Spec 20), Otolaryngology (Spec 21), Plastic surgery (Spec 23), Psychiatry (Spec 24 + 26), Dentist and Dental hygienist (Spec 49 + 50), Physiotherapist (Spec 51, 57, 62 and 65), Chiropodist (Spec 54 + 55 + 59 + 60), Chiropractor (Spec 53 and spec 64), Psychologist (Spec 63), General practitioner - all face-to-face contacts (Spec 80 and spec 81 + 82 + 83 +85 + 86 + 87 + 88 + 89 (out-of-hour medical service)).  **2. Number of musculoskeletal face-to-face contacts:** Anesthesiology (Spec. 01) (encounters regarding pain management), Diagnostic radiology (Spec 03 + 05) (encounters regarding musculoskeletal diagnosis), Rheumatology (Spec 06) (all encounters), Orthopedic surgery (Spec 20) (all encounters),  Physiotherapist (Spec 51) (musculoskeletal encounters), Chiropractor (Spec 53 and spec 64 (special clinical pathways for lumbar disc herniation, cervical disc herniation and lumbar spinal stenosis)), General practitioner (Spec 80) (musculoskeletal encounters based on algorithm (Appendix B). | Primary health care costs will be based on public subsidy for each service. Out of pocket charges for different health care services will not be considered. |
| **The Rehabilitation According to “The Danish Act of Health §140” register (Rehab-register)** | Contacts will be defined as a face-to-face encounter. Each course may have several contacts but only one contact each day will be considered. | **1 Number of all primary health care face-to-face contacts:**  All visits at a municipality rehabilitation unit  **2. Number of musculoskeletal face-to-face contacts:**  Number of contacts in a clinical course at a municipality rehabilitation unit beginning no later than 2 months after a hospital discharge registered with a musculoskeletal ICD-10 diagnostic code (see NPR: A and B diagnostic codes (ICD-10) considered to be musculoskeletal contacts). | Calculated expense per hour based on staff salaries and other operating costs for all municipality rehabilitation settings. |
| **The Diagnoses Related Group (DRG) Grouped National Patient Register** | Costs related to in- and outpatient contacts and emergency department visits in the NPR will be summoned per year based on diagnosis-related group (DRG) tariffs for all NPR activity. DRG-grouped NPR data itemizes payment instances and rates in the Danish health care system. Estimated grouped rates for each hospital service are based on the average costs for all hospitals in Denmark. | No contact data derived from DRG. | All secondary health care costs summoned for each follow up year. |
