# Supplementary material for: Number of musculoskeletal pain sites leads to increased long-term healthcare contacts and healthcare related costs – a Danish population-based cohort study
Source: BMC Health Serv Res. 2021 Sep 17;21:980. doi: 10.1186/s12913-021-06994-0 (PMC8447684; doi:10.1186/s12913-021-06994-0)
Supplement: Supplementary file 2 — Additional file 2. [file 12913_2021_6994_MOESM2_ESM.docx]

**Appendix B: Algorithm to identify MSK-contacts at General practitioners at The National Health Insurance Service Register**

To identify the number of face-to-face musculoskeletal health care contacts at General Practitioners (GP), the unique civil registration number (CPR number) (Mainz, Hess, & Johnsen, 2019; Pedersen, 2011; Schmidt, Pedersen, & Sorensen, 2014) assigned to all residents of Denmark was used to link individuals data from the medical records to register data from the National Health Insurance Service Register (HISR) (Andersen, Olivarius Nde, & Krasnik, 2011), the National Patient Register (NPS) (Lynge, Sandegaard, & Rebolj, 2011; Schmidt et al., 2015) and the Register of Medicinal Product Statistics (Johansen, Stenzhorn, Rosenzweig, Thirstrup, & Gazerani, 2013). The medicinal products register includes information about type of medication, price and prescriber of medicines sold on prescription and over-the-counter, as well as medication used by hospitalized patients. Information about sales of medicinal products in Denmark has been recorded since 1994 (Johansen et al., 2013; The Danish Health Data, 2016; Thygesen, Daasnes, Thaulow, & Bronnum-Hansen, 2011).

To determine if a face-to-face GP contact in the National Health Insurance Service Register was related to a musculoskeletal disorder a simple algorithm was developed. The algorithm built on available information from the National Health Insurance Service Register about each face-to-face GP contact, as well as information about subsequent health care activities from the National Health Insurance Service Register, the National Patient Register and the Register of Medicinal Product Statistics. Each face-to-face GP contact was evaluated in two steps. First, all activity codes for each face-to-face GP contact were evaluated. Those activity codes are supplementary administrative codes registered by GP at each face-to-face contact. According to the agreement between The Danish GP organization and the Danish Health Authority, activity codes initiate GP remuneration for services or activities taken at each contact. Such services could be specific diagnostic tests, laboratory tests such as B-hemoglobin (activity code 7108), C-reactive protein (CRP) (activity code 7120), strep-A test (activity code 7109), spirometry/peak flow (activity codes 7113, 7121, 7183) urinary stick (activity code 7101) or blood tests (activity codes 2601 and 2101). Face-to-face GP contacts were considered musculoskeletal contacts if they included activity codes 2109 (immobilizing bandages), 2111 (small fractures or relocations of small joints), 2119 (draining of liquid from joints), 2122 (first treatment – large fractures), 2123 (relocations of larger joints). In the second step, a face-to-face GP contact followed by primary care physiotherapy or chiropractor care seeking (within two months), collection of prescribed pain medication (within one month) or secondary health care seeking due to musculoskeletal disorders (within two months) were considered musculoskeletal contacts. All analyses were preformed using STATA 15.1 (StataCorp, College Station, Tx, USA).

| Algorithm to identify face-to-face GP (spec. 80) contacts (0101) from The National Health Service Register related to a musculoskeletal disorder | | |
| --- | --- | --- |
| Original HISR-data | Step 1.  Exclusion of GP contacts registered with non-musculoskeletal service or activity codes | Step 2.  Exclusion of GP contacts not followed by: |
| All face-to-face General Practitioners (GP) contacts (0101) from The National Health Insurance Service | 1. § 75 laboratory tests (7000 codes) 2. § 70 supplementary service codes (2000 codes) (except musculoskeletal codes: 2109,2111,2119,2122,2123) 3. Vaccine, child- and pregnancy examination codes (8000 codes) | 1. Physiotherapy contact (Primary care) within the following two months (HISR-data) 2. Collection or prescribed pain medication within the following month (RMPS-data) 3. In- or out-patient hospital contact for an MSK disorder within the following two months (NPS-data) |

## References

Andersen, J. S., Olivarius Nde, F., & Krasnik, A. (2011). The Danish National Health Service Register. *Scandinavian Journal of Public Health, 39*(7 Suppl), 34-37.

Johansen, A. N., Stenzhorn, A. A., Rosenzweig, M., Thirstrup, S., & Gazerani, P. (2013). Prescribing patterns and safety monitoring of duloxetine using the Danish Register of Medicinal Product Statistics as a source. *Scandinavian Journal of Public Health, 41*(8), 866-873.

Lynge, E., Sandegaard, J. L., & Rebolj, M. (2011). The Danish national patient register. *Scandinavian Journal of Public Health, 39*(7_suppl), 30-33.

Mainz, J., Hess, M. H., & Johnsen, S. P. (2019). The Danish unique personal identifier and the Danish Civil Registration System as a tool for research and quality improvement. *International journal for quality in health care : journal of the International Society for Quality in Health Care*.

Pedersen, C. B. (2011). The Danish civil registration system. *Scandinavian Journal of Public Health, 39*(7_suppl), 22-25.

Schmidt, M., Pedersen, L., & Sorensen, H. T. (2014). The Danish Civil Registration System as a tool in epidemiology. *European journal of epidemiology, 29*(8), 541-549.

Schmidt, M., Schmidt, S. A., Sandegaard, J. L., Ehrenstein, V., Pedersen, L., & Sorensen, H. T. (2015). The Danish National Patient Registry: a review of content, data quality, and research potential. *Clinical epidemiology, 7*, 449-490.

The Danish Health Data, A. (2016). Register of Medicinal Product Statistics (Vol. 2019).

Thygesen, L. C., Daasnes, C., Thaulow, I., & Bronnum-Hansen, H. (2011). Introduction to Danish (nationwide) registers on health and social issues: structure, access, legislation, and archiving. *Scandinavian Journal of Public Health, 39*(7 Suppl), 12-16.
