# Supplementary material for: Number of musculoskeletal pain sites leads to increased long-term healthcare contacts and healthcare related costs – a Danish population-based cohort study
Source: BMC Health Serv Res. 2021 Sep 17;21:980. doi: 10.1186/s12913-021-06994-0 (PMC8447684; doi:10.1186/s12913-021-06994-0)
Supplement: Supplementary file 3 — Additional file 3. [file 12913_2021_6994_MOESM3_ESM.docx]

**Appendix C: Directed Acyclic Graphs**

The purpose of this study is to test causal association. This demands carefully considerations regarding the choice of variables and the hypothesized structure between those variables and pathways(1). Directed acyclic graphs (DAGs) offer a systematic graphical representations of such causal relationships and hypothesized covariate structure (2). In this study, each research question has its own theoretical framework based on previous literature, theoretical considerations about the potential target trial and discussions within the author team. Choice of co-variables for statistical adjustment for each research question has been guided by the principle of minimal sufficient adjustment sets of co-variables for estimating the total effect (2, 3). In this approach, we only attempt to adjust for open ‘backdoor’ paths in the association between exposures and outcomes.

### Number of musculoskeletal pain sites

**Closed backdoor paths**

We hypothesized that the causal relationship between number of musculoskeletal pain sites and healthcare utilization is mediated through factors like health-related quality of life, fear avoidance beliefs, catastrophizing (unmeasured variable) and activity evoked pain. This creates closed backdoor paths without a need for adjustment.

**Open backdoor paths**

We consider duration of pain to be associated with number of musculoskeletal pain sites and associated with healthcare use (4, 5). In building this causal model, we took the position that duration of pain creates an open backdoor pathway in the association between number of musculoskeletal pain sites and healthcare utilization with need for adjustment.

We consider comorbidity to be associated with number of musculoskeletal pain sites as number of musculoskeletal pain sites and number of non-musculoskeletal symptoms increase proportionally (6) and comorbidity is in itself a driver for healthcare seeking (7). Sex and age are non-modifiable factors. None of these factors are considered the cause of pain, still, both are associated with number of musculoskeletal pain sites, as women and older people report chronic and widespread pain more often than men/younger people (5). Furthermore, women and older people seek healthcare more often than men/younger people (8). Higher level of education is not a cause of musculoskeletal pain but higher level of education is associated with better general health and seems to protect against development of musculoskeletal pain and people with higher education seek healthcare less often. This relationship could work through factors like health literacy and self-efficacy (unmeasured variables), job satisfaction etc. Personality traits and health anxiety may influence the development of musculoskeletal pain and coping strategies. This relationship might work through pathways like harm avoidance, being fearful, being pessimistic, high dependence on reassurance, low level of motivation and meaningful personal goal setting (9). Especially the personality trait neuroticism has been suggested as an important factor in development of musculoskeletal pain and ability to cope with it (9). We consider depressive symptoms (feeling down and sad) to be associated with increasing number of musculoskeletal pain sites (10) and to influence healthcare seeking. Physical work exposures are associated with musculoskeletal pain. High level of work exposure might influence the development of musculoskeletal pain and pain-related disability. Therefore, we consider sex, age, health anxiety, duration of pain, level of education, comorbidity, personality traits (extraversion, agreeableness, conscientiousness, neuroticism and openness), risk of depression, marital status, physical job exposure and previous healthcare utilization to be confounders of the relationship between number of musculoskeletal pain sites and care seeking, and therefore adjustment is needed.

**Other variables**

High pain intensity is associated with higher healthcare utilization. In this study we wanted to analyse the consequences of number of musculoskeletal pain sites with non-trivial pain on healthcare utilization. Therefore, we chose to incorporate data on pain intensity in the `number of musculoskeletal pain sites´ variable. Factors like cause of pain, leisure time physical activity, type of pain (pain mechanism), musculoskeletal diagnosis could potentially be confounders in this study, but these data were not available.


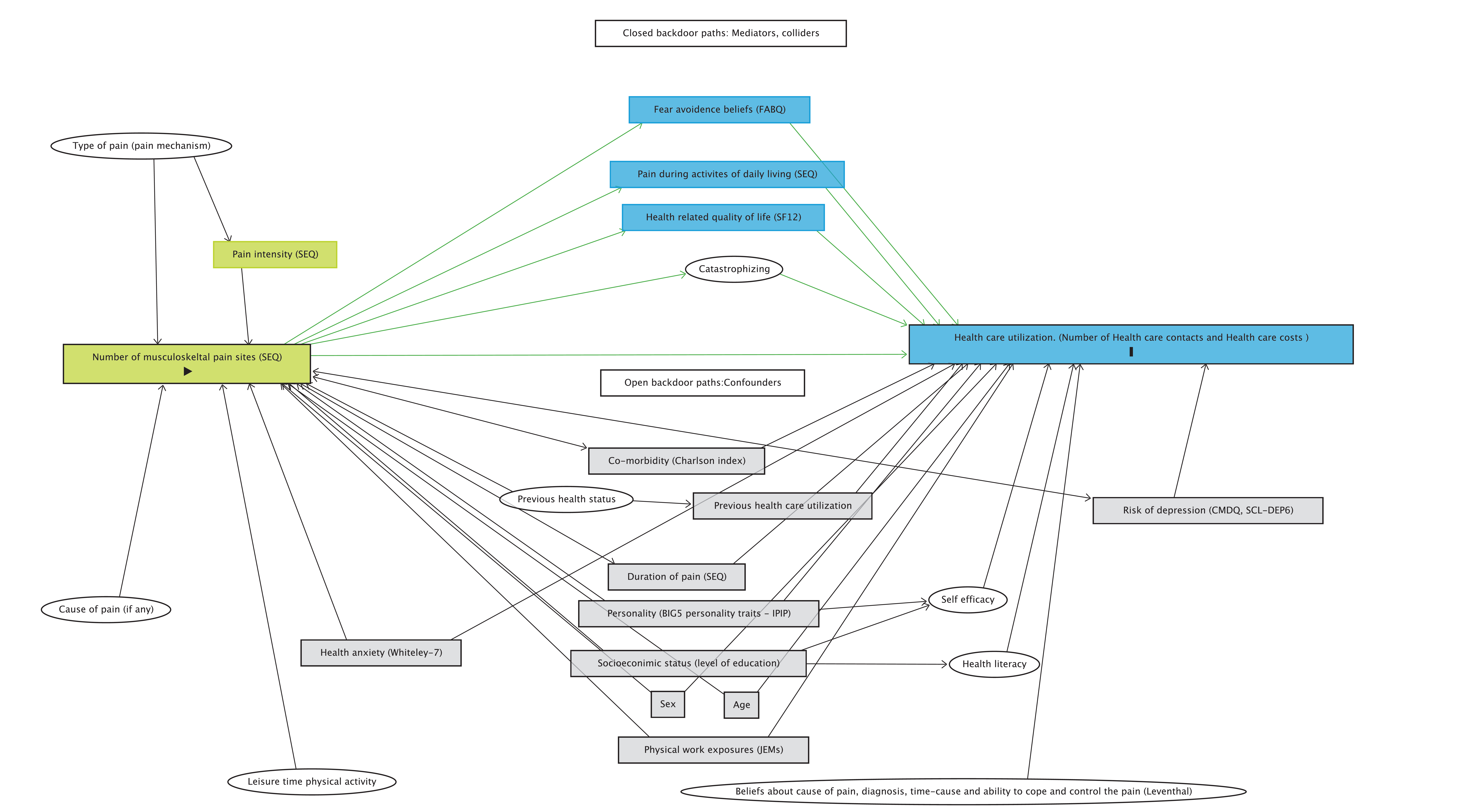


**Figure 1. Hypothesized causal relationships between number of musculoskeletal pain-sites and healthcare contacts/costs illustrated via Directed Acyclic Graphs (**[**www.dagitty.net**](http://www.dagitty.net)**)**


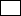
 Headline for groups of covariables.  exposure.
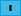
 outcome.
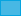
 mediator/collider.
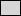
  adjusted variable/confounders.
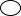
 unmeasured variable.
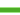
 causal/closed backdoor paths. confounding/open backdoor paths.


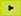


Arrows indicate hypothezied direction of relationships. Relationships between covariables in the DAG is not illustrated.

### Health anxiety

**Closed backdoor paths**

We hypnotized that the causal relationship between health anxiety and healthcare utilization is mediated through factors like health-related quality of life, depressive symptoms/mental health, fear avoidance beliefs, catastrophizing (unmeasured variable), engagement in preventive strategies (unmeasured variable), information seeking/reassurance (unmeasured variable) and beliefs about pain. These factors interrelate in complex bidirectional relationships (not illustrated). This creates closed backdoor paths without need for adjustment.

**Open backdoor paths**

We consider comorbidity to be associated with health anxiety in a bidirectional relationship and comorbidity is in itself a driver for healthcare seeking (7). Sex and age are non-modifiable factors. Neither are considered the cause of health anxiety but we consider both factors to be associated with health anxiety, and women and older people seek healthcare more often than men/younger people. People with higher level of education seek healthcare less often. This relationship could work through factors like health literacy and self-efficacy (unmeasured variables), job satisfaction etc. We consider lower levels of education to be associated with higher level of health anxiety and higher levels of care seeing. Personality traits may influence the development health anxiety and coping strategies, including care seeking. This relationship might work through pathways such as a tendency to be fearful, pessimistic or high dependency of reassurance, because individuals with health anxiety are likely to exaggerate negative information or attend to information that supports their health concerns (11). We consider health anxiety to be more prevalent among individuals with low level of social support and low level of social support to be associated with care seeking. Pain characteristics like increasing number of musculoskeletal pain sites and duration of pain could influence both health anxiety and care seeking. This means that sex, age, duration of pain and number of musculoskeletal pain sites, level of education, comorbidity, personality traits (extraversion, agreeableness, conscientiousness, neuroticism and openness), marital status/social support and previous healthcare utilization create open backdoor pathways in the relationship between health anxiety and care seeking and adjustment is needed.

##
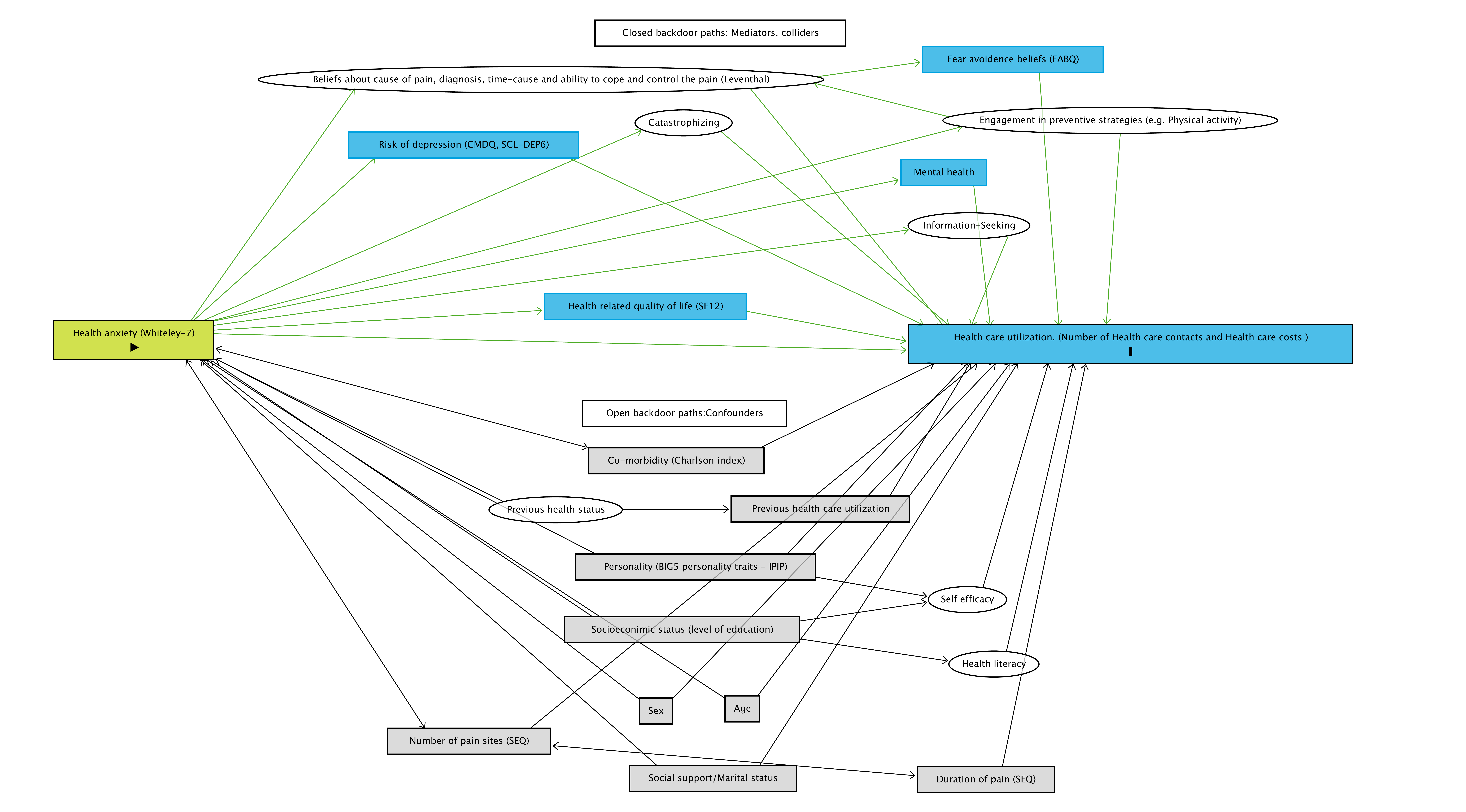


**Figure 2. Hypothesized causal relationships between health anxiety and healthcare contacts/costs illustrated via Directed Acyclic Graphs (**[**www.dagitty.net**](http://www.dagitty.net)**)**

 Headline for groups of covariables.
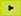
 exposure.
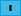
 outcome.
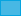
 mediator/collider.
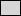
  adjusted variable/confounders.
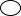
 unmeasured variable.
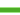
 causal/closed backdoor paths. confounding/open backdoor paths.


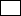


Arrows indicate hypothesized direction of relationships. Relationships between covariables in the DAG is not illustrated.

### References

1. Lederer DJ, Bell SC, Branson RD, Chalmers JD, Marshall R, Maslove DM, et al. Control of confounding and reporting of results in causal inference studies. Guidance for authors from editors of respiratory, sleep, and critical care journals. Annals of the American Thoracic Society. 2019;16(1):22-8.

2. Textor J, van der Zander B, Gilthorpe MS, Liśkiewicz M, Ellison GT. Robust causal inference using directed acyclic graphs: the R package ‘dagitty’. International journal of epidemiology. 2016;45(6):1887-94.

3. Knüppel S, Stang A. DAG Program:: Identifying Minimal Sufficient Adjustment Sets. Epidemiology. 2010;21(1):159.

4. IsHak WW, Wen RY, Naghdechi L, Vanle B, Dang J, Knosp M, et al. Pain and Depression: A Systematic Review. Harv Rev Psychiatry. 2018;26(6):352-63.

5. Fayaz A, Croft P, Langford RM, Donaldson LJ, Jones GT. Prevalence of chronic pain in the UK: a systematic review and meta-analysis of population studies. BMJ Open. 2016;6(6):e010364.

6. Tschudi-Madsen H, Kjeldsberg M, Natvig B, Ihlebaek C, Dalen I, Kamaleri Y, et al. A strong association between non-musculoskeletal symptoms and musculoskeletal pain symptoms: results from a population study. BMC musculoskeletal disorders. 2011;12:285-2474-12-285.

7. Fayaz A, Ayis S, Panesar SS, Langford RM, Donaldson LJ. Assessing the relationship between chronic pain and cardiovascular disease: A systematic review and meta-analysis. Scand J Pain. 2016;13:76-90.

8. Ferreira ML, Machado G, Latimer J, Maher C, Ferreira PH, Smeets RJ. Factors defining care-seeking in low back pain--a meta-analysis of population based surveys. European journal of pain (London, England). 2010;14(7):747.e1-.e7.

9. Naylor B, Boag S, Gustin SM. New evidence for a pain personality? A critical review of the last 120 years of pain and personality. Scand J Pain. 2017;17:58-67.

10. Christensen JO, Johansen S, Knardahl S. Psychological predictors of change in the number of musculoskeletal pain sites among Norwegian employees: a prospective study. BMC musculoskeletal disorders. 2017;18(1):1-13.

11. Eastin MS, Guinsler NM. Worried and wired: effects of health anxiety on information-seeking and health care utilization behaviors. CyberPsychology & Behavior. 2006;9(4):494-8.
